# Delayed γH2AX foci disappearance in mammary epithelial cells from aged women reveals an age-associated DNA repair defect

**DOI:** 10.18632/aging.101849

**Published:** 2019-03-14

**Authors:** Teresa Anglada, Joan Repullés, Anna Espinal, Mark A LaBarge, Martha R Stampfer, Anna Genescà, Marta Martín

**Affiliations:** 1 Department of Cell Biology, Physiology and Immunology, Universitat Autònoma de Barcelona, 08193 Bellaterra, Spain; 2 Microscopy Platform, Biomedical Research Institute Sant Pau (IIB-Sant Pau), 08041Barcelona, Spain; 3 Servei d’Estadística Aplicada, Universitat Autònoma de Barcelona, 08193 Bellaterra, Spain; 4 Department of Population Sciences, and Center for Cancer and Aging, Beckman Research Institute at City of Hope, Duarte, CA 91010, USA; 5 Biological Systems and Engineering Division, Lawrence Berkeley National Laboratory, Berkeley, CA 94720, USA; * Equal contribution

**Keywords:** aging, double-strand break repair, γH2AX, human mammary epithelial cells, DNA damage, genome integrity

## Abstract

Aging is a degenerative process in which genome instability plays a crucial role. To gain insight into the link between organismal aging and DNA repair capacity, we analyzed DNA double-strand break (DSB) resolution efficiency in human mammary epithelial cells from 12 healthy donors of young and old ages. The frequency of DSBs was measured by quantifying the number of γH2AX foci before and after 1Gy of γ-rays and it was higher in cells from aged donors (ADs) at all times analyzed. At 24 hours after irradiation, ADs retained a significantly higher frequency of residual DSBs than young donors (YDs), which had already reached values close to basal levels. The kinetics of DSB induction and disappearance showed that cells from ADs and YDs repair DSBs with similar speed, although analysis of early times after irradiation indicate that a repair defect may lie within the firing of the DNA repair machinery in AD cells. Indeed, using a mathematical model we calculated a constant factor of delay affecting aged human epithelial cells repair kinetics. This defect manifests with the accumulation of DSBs that might eventually undergo illegitimate repair, thus posing a relevant threat to the maintenance of genome integrity in older individuals.

## Introduction

The aging process is related to a loss of function and an increased probability of developing several diseases, such as cancer. Cellular changes associated with aging are an accentuated inflammatory response, alterations in the redox cellular equilibrium, telomere attrition, and changes in nuclear structure, and all of them imply relevant threats to maintenance of genomic integrity [1].

Of the many lesions that DNA can suffer, the DNA double strand break (DSB) poses a considerable threat because joining of illegitimate ends can occur. One of the earliest events in DSB signaling is the phosphorylation of the histone H2AX on serine 139, γH2AX [2]. Phosphorylation of H2AX spreads over megabases surrounding the break site, resulting in a platform that enables the recruitment of effector proteins at the damaged DNA [3]. The modification of H2AX can be identified as discrete foci forming at DSB sites and scoring of γH2AX foci is a widely used tool to estimate the number of DSBs induced after exposure to damaging agents [4]. γH2AX foci disappearance over time is a good approach to assess kinetics of DSB repair because once DNA has been repaired, H2AX phosphorylation disappears and foci are no longer detectable [5]. The DSB repair kinetics follow a biphasic pattern: most of the DSBs are repaired by the fast component of repair within the first two hours after induction, while the remaining DSBs can be repaired by the slow component of repair, which acts with slower kinetics and might require several hours –or even days– to complete repair [6–8].

Studies with models of *in vitro* aging have provided evidence of a higher frequency of unrepaired DSBs with time in culture. For example, replicative senescent cells accumulate more γH2AX than dividing cells, suggesting a reduced repair ability or accumulation of DNA damage associated with replicative halt [9]. Also, non-senescent late population doubling (PD) cells during *in vitro* culture present with more unrepaired DSBs and more γH2AX signaling than earlier PD cells [10,11]. A similar tendency is observed with organismal aging, as cells from aged human donors present with an increased frequency of chromosomal reorganizations and γH2AX foci with increasing age [11–14]. Although the increased frequency of DSBs with age is clear, the mechanisms underlying it are yet unknown.

The presence of a greater number of lesions in the DNA of aged cells could be due to a progressive accumulation of lesions over time, to difficult-to-repair DSBs marked by persistent γH2AX foci or to a limited capacity of aged cells to repair new DSBs [15–17]. The general notion of declined DSB repair efficiency with age is supported by some studies. Accumulation of residual γH2AX foci after *ex vivo* ionizing irradiation (IR) exposure of fibroblasts and hematopoietic stem cells of healthy donors suggests that older individuals have a reduced DSB repair capacity [14,18]. Similarly, Garm and colleagues [19] used comet assays and flow cytometry techniques to measure DSBs in peripheral blood mononuclear cells from twins who ranged from 40 to 77 years of age, and observed a tendency towards diminished DSB repair with increasing age. In contrast, human dermal fibroblasts from aged donors showed a heterogeneous capacity for DSB repair after analyzing γH2AX fluorescence intensity [12], and even an increased DSB repair rate with age in lymphocytes from 94 donors exposed to IR [20]. Therefore, although the collected evidence suggests that the frequency of DNA-DSBs increases with age in multiple mammalian tissues, the DSB repair capacity of cells from aged individuals is still controversial and the mechanisms underlying age-related DSB accumulation remain unclear.

To gain insight into the consequences of organismal aging on DNA damage repair capacity, we have measured DSB induction and resolution in finite lifespan non-transformed (pre-stasis) human mammary epithelial cells (HMECs) from 12 female donors of young (≤ 27) and old (≥ 60) ages. Our work shows that cells from aged women have a higher basal level of DSBs and display a sharp decline of DSB repair efficiency that leads to the accumulation of these lesions after exposure to low doses of IR. Both, observed data and mathematical modelling of DSB repair kinetics indicate that old donors display a delayed firing of the DNA damage response that contributes to the accumulation of damage with age.

## RESULTS

### Defining the criteria for analyzing DNA double strand breaks in pre-stasis HMECs

**Figure 1:**
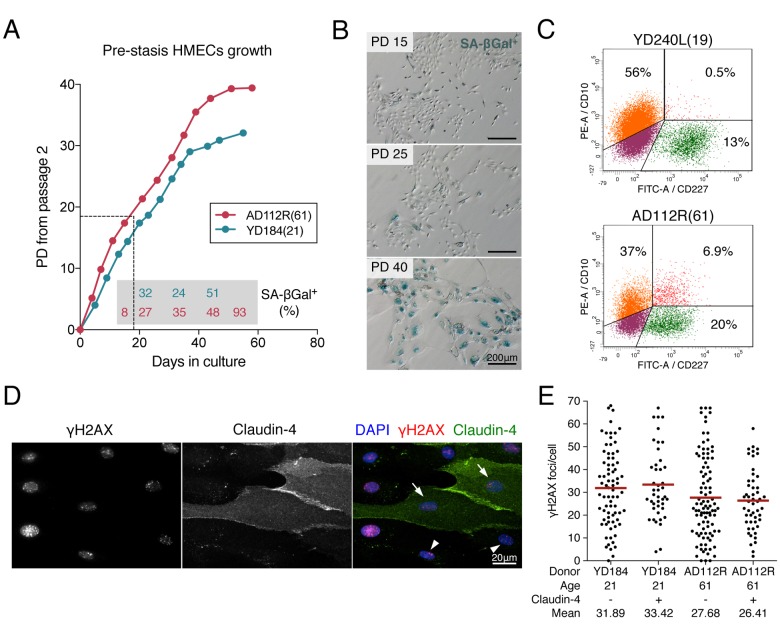
**Pre-stasis HMEC characterization and culture.** (**A)** Representative growth curves of HMECs from YD184(21) and AD112R(61) in M87A medium with supplements. Dots correspond to correlative cell passages from passage 2. The dotted thin line indicates the early passages used for the experiments. Percentages of SA-β-Gal positive cells are indicated within the grey box (*N* > 500 cells). (**B)** The frequency of SA-β-Gal positive cells increases with time in culture. (**C)** Diagrams of flow cytometry analysis of CD10 (PE, phycoerytrin) and CD227 (FITC, fluorescein isothiocyanate) in YD240L(19) and AD112R(61) (*N* > 10000 cells). (**D)** Images of the immunofluorescent staining of claudin-4 (expressed by luminal cells, FITC, green), γH2AX (Cy3, red) and DAPI (blue) at 2h after 1Gy of γ-rays exposure. Claudin-4 positive (arrows) and negative (arrowheads) cells are shown. (**E)** Scatter dot plot and average number (red line) of γH2AX foci/cell in claudin-4 positive and negative cells (*N* > 100 cells/donor). No statistical differences were observed (Mann-Whitney test, *p*-value > 0.05).

HMECs were obtained from reduction mammoplasty tissue of 12 donors, which were classified according to age into young donors (YDs, ≤ 27, age in parentheses): YD48R(16), YD240L(19), YD168R(19), YD184(21), YD59L(23) and YD123(27) and aged donors (ADs, ≥ 60, age in parentheses): AD153L(60), AD112R(61), AD122L(66), AD29(68), AD429ER(72), AD353P(72). Cells were cultured as pre-stasis strains in M87A medium as described by Garbe and colleagues [21], to support their long-term growth (Figure 1A). Despite using a low-stress medium, there was an accumulation of senescent cells with time in culture (Figure 1A and 1B). In order to avoid interference from replicative-senescence associated DNA damage when assessing age-dependent differences in the formation and resolution of DSBs, early PDs were chosen (PD < 20 which correspond to passages 4^th^ to 6^th^) in which the frequency of senescent cells was ≤ 10%.

As previously reported [21,22] we found age-related differences in the fractions of myoepithelial (CD10^+^/CD227^-^) and luminal (CD10^-^/CD227^+^) cells in HMEC culture. Flow cytometry analysis of CD10 and CD227 cell-lineage specific markers confirmed an age-dependent decrease in the myoepithelial fraction accompanied by an increase of the luminal fraction (CD10^+^/CD227^-^ in YD240L: 56.05%; AD112R: 37.29%; CD10^-^/CD227^+^ in YD240L: 12.67%; AD112R: 20.06%) (Figure 1C). In order to rule out radiation-sensitivity differences between the two breast cell types, cells from young and aged donors were exposed to 1Gy of γ-rays and labelled with γH2AX and claudin-4 (Cl4), a cytoplasmic membrane protein mostly expressed by luminal cells (Figure 1D). As shown in Figure 1E, there were no differences in the frequency of γH2AX foci between Cl4^+^ and Cl4^-^ cells 2h after irradiation in any of the donors analyzed (Cl4^-^: 31.89 and Cl4^+^: 33.42 in the YD184; Cl4^-^: 27.68 and Cl4^+^: 26.41 in the AD112R; Mann-Whitney test, *p*-value > 0.05). These results indicate that radiation-induction of DSBs is similar in myoepithelial and luminal HMECs, ruling out the need to distinctively identify them when analyzing age-dependent differences in DNA repair.

### Mammary epithelial cells from aged donors show an increased basal frequency of DSBs

**Figure 2:**
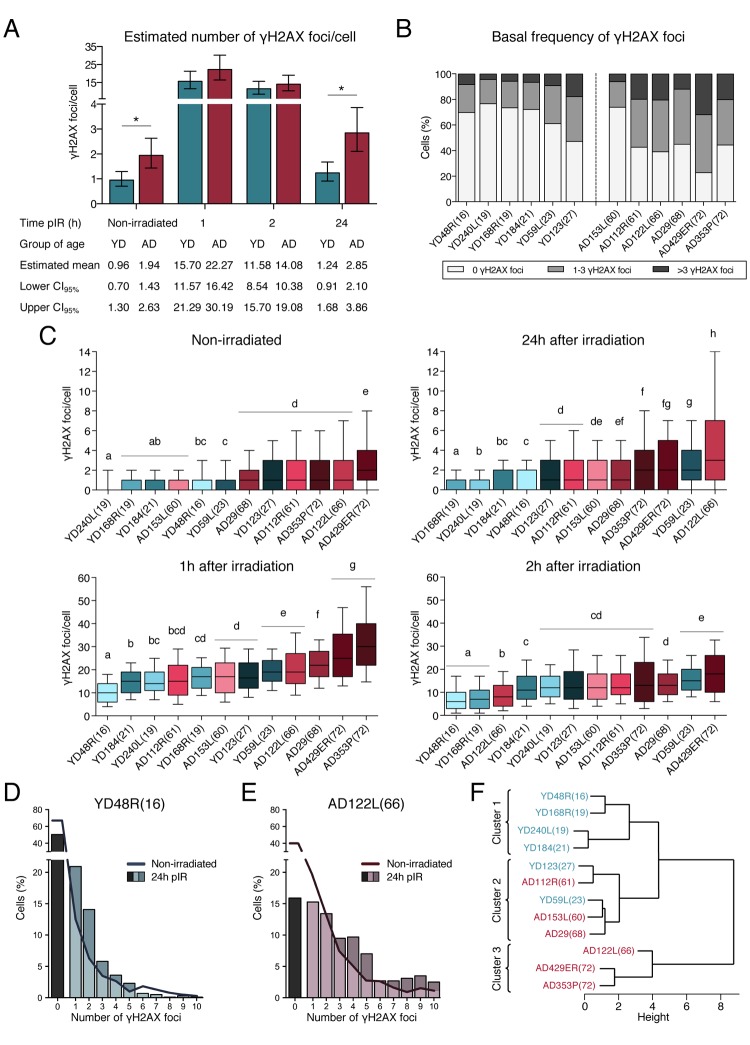
**Frequencies of γH2AX foci in HMECs from young and aged donors.** (**A**) Estimated mean number of γH2AX foci/cell and confidence intervals for young and aged donors. Asterisks indicate significant differences between YDs and ADs (generalized linear model, *p*-value < 0.01). The number of cells analyzed for each donor is stated in Table 1. (**B**) Frequency of cells with a defined number of γH2AX foci in non-irradiated samples from the 12 donors. The number of cells analyzed for each donor is stated in Table 1. (**C**) Box plots of the frequency of γH2AX foci in cells from YDs and ADs in non-irradiated samples and at 1h, 2h or 24h after exposure to 1 Gy of γ-rays. Each donor is colored with blue or red depending on the group of age (blue for YDs and red for ADs). In each group, colors become darker with increasing age of the donor. Boxes include data from the upper to the lower quartile. The median is represented with a black line and whiskers compile 10 to 90% of the scored values. The number of cells analyzed for each donor is stated in Table 1. Statistical differences between donors are indicated following a letter code: donors signaled with the same letter do not show statistical differences and therefore different letters indicate statistically significant differences between donors (Kruskal-Wallis test with Dunn’s multiple comparisons correction, *p*-value < 0.05). (**D, E**) Distribution of cells according to the number of γH2AX foci/cell individually scored in YD48R(16) (**D**) and in AD122L(66) (**E**). Bars indicate the percentage of cells without foci (black bar) or with ≥1 γH2AX foci (colored bars) 24h after irradiation. The continuous line depicts this percentage before irradiation. The number of cells analyzed for each donor is stated in Table 1. (**F**) Hierarchical clustering of the 12 donors according to the standardized mean number of γH2AX foci scored in non-irradiated samples and at 1, 2 and 24h after IR. The number of cells analyzed for each donor is stated in Table 1.

γH2AX foci are accepted as surrogate markers of DSBs [23], but the pattern of γH2AX staining and the number of foci scored are dependent on the phase of the cell cycle analyzed (Supplementary Figure 1). To mitigate variability due to cell cycle, γH2AX foci counting was restricted to cells in G1 phase, which were identified by pericentrin labelling, a centrosomal protein that duplicates along with DNA, allowing clear distinction of cell cycle phase for each individual cell analyzed [24]. γH2AX foci were scored before and after exposure of HMECs to IR (1h, 2h and 24h pIR). In order to detect differences in γH2AX foci numbers between age groups (young donors *vs* old donors), a generalized linear model with repeated measures for each donor was established (see Materials and Methods section). We first established the basal frequency of DSBs in cells from young and old donors, in non-irradiated samples. Using the generalized linear model, the estimated mean number of γH2AX foci before irradiation was 0.96 (CI_95%_ = [0.70, 1.30]) in cells from YDs and 1.94 (CI_95%_ = [1.43, 2.63]) in cells from ADs, twice the level scored in YDs (Figure 2A). Statistically significant differences between basal γH2AX foci frequencies in YDs and ADs were detected (*p*-value = 0.0013; t = -3.22). In addition to a lower basal frequency of DSBs, in most young donors (5 out of 6) 60%-75% of cells were devoid of any γH2AX foci, whereas in most old donors (5 out of 6) less than 45% of cells were devoid of foci (Figure 2B). In addition, in most YDs less than 10% of cells carried more than 3 γH2AX foci per cell, whereas ~20% of cells from ADs had more than 3 foci (X^2^ test, *p*-value < 0.0001). Despite the existence of inter-individual differences between donors of similar ages, these analyses demonstrate that both, the average basal frequency of DSBs and the fraction of cells carrying DSBs are higher in HMECs from aged donors as compared to young donors.

Descriptive statistics was computed for each donor (Table 1 and Supplementary Figure 2) and statistical differences regarding the mean number of γH2AX foci per cell of each donor were calculated (Kruskal-Wallis test with a Dunn’s multiple comparisons correction). When donors were lined up based on statistical differences among them, most of YDs and ADs aligned according to an age-dependent order (Figure 2C Non-irradiated). This analysis allowed us to detect that unirradiated cells from YD123(27) and AD153L(60) did not behave as the rest of the donors of their age group (Table 1, Figure 2C Non-irradiated), thus unmasking the existence of inter-individual differences among donors. Besides these particular exceptions, the rest of YDs had a similar and low frequency of basal DSBs/cell (Table 1 and Figure 2C Non-irradiated) and, consequently, they statistically grouped together (a, b and c) and were significantly different from most of ADs (d, e), which carry more basal DSBs/cell and display a greater data dispersion.

**Table 1:** Descriptive analysis of the number of γH2AX foci per cell.

**Donor**	**Non-irradiated**			**1h post-irradiation**				**2h post-irradiation**						**24h post-irradiation**				
	**Mean**	**SD**	**N**		**Mean**	**SD**	**N**	**Mean**		**SD**		**N**		**Mean**		**SD**		**N**
**Young donors (YDs)**	**0.90**	**2.52**	**6152**		**16.16**	**7.85**	**2777**		**11.43**		**8.22**		**4046**	**1.41**	**2.33**		**5633**	
YD48R(16)	0.91	2.17	957		10.63	5.94	464		7.64		7.04		923	1.21	2.04		1000	
YD240L(19)	0.71	2.64	1975		15.42	7.32	389		13.25		7.62		685	0.87	1.87		1000	
YD168R(19)	0.78	2.60	809		17.13	7.34	357		8.05		6.38		740	0.58	1.40		879	
YD184(21)	0.79	2.13	1000		15.04	6.89	470		12.87		8.02		752	1.05	1.72		1000	
YD59L(23)	1.05	2.21	1000		19.75	7.22	609		16.17		7.15		511	2.87	2.75		956	
YD123(27)	1.93	3.64	411		17.9	8.60	488		14.30		9.91		435	1.98	3.10		798	
**Aged donors (ADs)**	**2.03**	**3.85**	**4702**		**21.17**	**11.85**	**3616**		**13.79**		**9.52**		**3800**	**2.77**	**4.62**		**4730**	
AD153L(60)	0.78	2.30	998		17.36	9.00	895		13.72		8.61		733	2.08	2.85		996	
AD112R(61)	2.20	3.57	770		16.31	9.75	780		14.18		8.34		671	2.33	5.18		996	
AD122L(66)	2.68	4.91	984		21.45	11.15	588		9.33		6.71		822	5.38	6.85		484	
AD29(68)	1.48	2.26	707		22.38	8.04	656		13.97		7.13		799	2.03	2.31		1000	
AD429ER(72)	3.43	4.57	483		27.72	13.31	361		19.14		11.82		252	3.10	3.83		353	
AD353P(72)	2.30	4.29	760		32.7	16.31	336		16.16		13.33		615	3.29	5.58		901	
												

### Aged donors accumulate higher levels of DSBs after irradiation

To study the efficiency of DSB repair with age, exponentially growing cell cultures from all donors were exposed to 1Gy of γ-rays. One hour after IR exposure, the estimated mean number of γH2AX/cell was 15.70 (CI_95%_ = [11.57, 21.29]) in YDs versus 22.27 (CI_95%_ = [16.42, 30.19]) in cells from ADs (Figure 2A). As shown in Table 1, at this time point the mean number of γH2AX foci per cell strongly correlated with the age of the donors, ranging from 10.63 γH2AX foci per cell in the youngest donor (YD48R(16)) to 32.7 γH2AX foci per cell in the oldest donor (AD353P(72)). Alignment of donors at this time according to statistics (Kruskal-Wallis test and Dunn’s multiple comparisons correction), rendered clear differences between young and old donors and most of them continued to maintain an age-related position (Figure 2C 1h after irradiation). Again, data from YDs showed little variance, revealing similar DSB repair efficiencies while ADs presented with more γH2AX foci and higher inter-cellular variability. Overall, 1h after irradiation cells from ADs accumulated higher levels of unrepaired DSBs, suggesting that these cells elicit a less efficient response from the fast component of DSBs repair.

When γH2AX foci were scored two hours after IR exposure the estimated mean number of γH2AX foci per cell had already decreased in all donors and it was similar for YDs (11.58 γH2AX foci/cell, CI_95%_ = [8.54, 15.70]) and for ADs (14.08 γH2AX foci/cell, CI_95%_ = [10.38, 19.08]) (Figures 2A and 2C 2h after irradiation, Table 1). The decline in γH2AX foci during this second hour was higher in cells from ADs than in cells from YDs, suggesting that the initial impairment in DSBs repair shown by ADs 1h after irradiation is eventually alleviated.

In order to evaluate the efficiency in the slow component of DNA repair, we finally analyzed the frequency of γH2AX foci 24 hours after IR exposure. Both YDs and ADs have repaired most of the radiation induced DSBs, but while most of the YDs had reached a frequency of residual DSBs close to the basal levels, only two aged donors had reached their basal levels of DSBs (Table 1). Thus, cells from ADs displayed a higher estimated mean number of γH2AX foci/cell than cells from YDs (YDs: 1.24 γH2AX foci/cell, CI_95%_ = [0.91, 1.68]; ADs: 2.85 γH2AX foci/cell, CI_95%_ = [2.10, 3.86]; *p*-value = 0.0001; t = -3.79) (Table 1, Figure 2A). Indeed, when donors were individually compared (Kruskal-Wallis test and Dunn’s multiple comparisons correction) the differences between YDs and ADs allowed a clear age-related alignment (Figure 2C 24h after irradiation). Not only YD cells present with less γH2AX foci/cell, but also the frequency of cells devoid of γH2AX foci at 24h is 50%, close to their frequency before irradiation (70%) (Figure 2D). In contrast, in ADs the frequency of cells without γH2AX foci at 24h after irradiation is far from their basal frequency (15% *vs* 40%) (Figure 2E). Among cells with γH2AX foci, most of the YDs’ cells scored only 1 or 2 γH2AX foci per cell at 24h pIR, whereas ADs still accumulated 3 or more γH2AX foci per cell (Figures 2D and 2E). Thus, at 24 hours after irradiation more cells from ADs accumulate DSBs, and also the frequency of DSBs per cell is higher than in YDs.

Finally, and in order to determine if the γH2AX foci disappearance was a good marker of chronological age, we carried out a hierarchical clustering analysis using standardized values of γH2AX foci from the 12 donors in the 4 time points (non-irradiated, 1h, 2h and 24h after IR). With these data, the donors were grouped in 3 clusters (Figure 2F). A clearly separated cluster was constituted by the oldest donors (AD122L(66), AD429ER(72) and AD353P(72)), which displayed the worst repair efficiency among all the donors. The 4 youngest donors (YD48R(16), YD168R(19), YD240L(19) and YD184(21)), which are the ones with the best DSB repair performance, clustered together and separated from the other donors. And finally, an intermediate cluster included the remaining young donors (YD123(27) and YD59L(23)) along with the 3 aged donors (AD153L(60), AD112R(61) and AD29(68)) that frequently did not follow an age-dependent order in the previous statistical analyses (Figure 2C). Hence, hierarchical clustering of donors according to γH2AX foci at different times after irradiation reveals that DSB repair efficiency is a good marker of age.

### Delayed firing of the DNA Damage Response (DDR) with age

**Figure 3:**
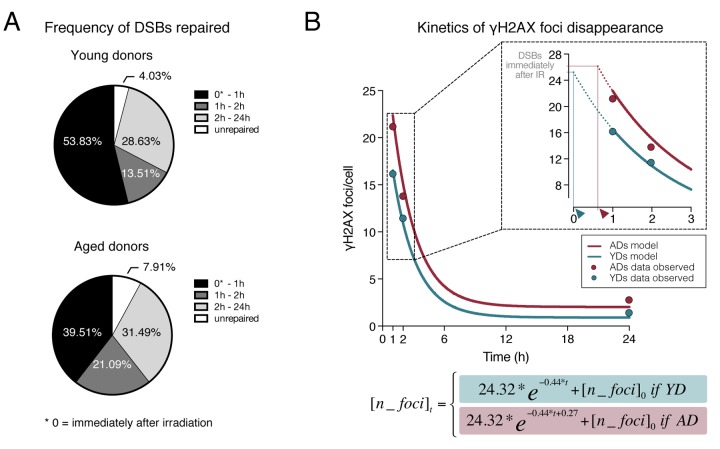
**Dynamics of γH2AX foci disappearance after irradiation.** (**A**) Frequency of DSBs repaired within defined time intervals after exposure to 1Gy of γ-rays for YDs and ADs. The number of DSBs induced after 1Gy exposure (𝜃) of G1 cells was estimated to be of 35 according to Rothkamm & Löbrich [25]. For the other time points, the numbers of DSBs are those depicted in Tables 1 and 2. (**B**) Kinetics of γH2AX foci disappearance for young and aged donors following the model of first order kinetic reaction stated in Materials and Methods section. The mean number of γH2AX foci scored at each time point is represented with dots (blue for YDs, red for ADs) and it is stated in Table 1. The lines represent the kinetics of DSBs repair estimated after modeling data of all γH2AX foci/cell from the 12 donors at 1, 2 and 24h after irradiation. The number of cells analyzed for each group of age is stated in Table 1. The inset in the graph shows a detail of the early times after IR exposure. The dotted lines represent an extrapolation of the DSB repair kinetics in the time interval comprised between the DSB repair initiation and 1h after IR. Arrowheads indicate the moment of repair initiation, when the extrapolation lines for YDs and ADs reach the number of γH2AX foci present immediately after IR.

DSB repair is not constant, as it follows biphasic exponential negative kinetics. In order to determine the nature of the repair defect displayed by cells from older donors, we aimed to describe the kinetics of DSB repair for the two age groups. We first calculated the rate of γH2AX foci disappearance for each time interval analyzed (Table 2). Because γH2AX foci assay does not allow the scoring of the DSBs induced immediately after irradiation (𝜃), to estimate γH2AX foci disappearance at the initial time interval we have used the previously described standard estimation of 35 DSBs induced per Gy of radiation in G1 cells [25]. According to this, during the first hour after DNA damage induction, the rate of DSB resolution was higher for YDs (53.83% of γH2AX foci disappeared) than for ADs (39.51%) (Figure 3A and Table 2), indicating a greater DSB repair ability for YDs immediately after DNA damage induction, while ADs end the 1^st^ hour carrying higher numbers of unresolved DSBs. In contrast, the rate of γH2AX foci disappearance between 1 and 2h after IR was higher in AD (21.09%) than in YD samples (13.51%). Two hours after irradiation ADs have repaired 60.6% of radiation-induced DSBs, very close to the 67.3% of DSBs repaired by YDs’ cells, suggesting that, although with some delay, cells from ADs are eventually able to launch the DDR and efficiently resolve the accumulated DSBs. The last time interval analyzed (2 to 24h post-irradiation) corresponds to the slow component of repair, in which the rates of DSBs repaired were very similar for both young (28.63%) and aged donors (31.49%) (Figure 3A and Table 2), suggesting that age-related differences in DNA repair efficiency lay within the initial times after DNA damage induction.

**Table 2:** Rate of DSBs repair within intervals of time.

**Time after IR exposure (h)**	**DSB induced/ remaining**		**Time interval**	DSB repaired (%) **	
	**YDs**	**ADs**		**YDs**	**ADs**
0*	35	35			
**1**	16.16	21.17	0* - 1h	53.83	39.51
**2**	11.43	13.79	**1h - 2h**	13.51	21.09
**24**	1.41	2.77	**2h - 24h**	28.63	31.49

* 0 = immediately after irradiation. The current estimation of 35 DSB per Gy is used [25].

** Frequency of DSB repair has been calculated as the difference in DSBs between one time point and the time immediately before, assuming that repairing 35 DSBs is 100% of repair.

In order to test the hypothesis of an age-related delay in DNA-DSB repair initiation, we established a first order kinetic reaction using a Nonlinear Regression Model. The model, described in Materials and Methods section, predicts the number of γH2AX foci in AD and YD cells and it is resolved as follows:

[n_foci]t=24.32* e-0.44 * t+ [n_foci]0 if YD24.32* e-0.44 * t+0.27+ [n_foci]0 if AD

The repair of DSBs is estimated by the constant of γH2AX foci decay β_1_ = -0.44, CI_95%_ = [-0.64, -0.25] for young and aged donors. In the case of ADs, the model includes a constant factor of delay in DSB repair initiation, which is estimated to be β_0_ = 0.27, CI_95%_ = [0.08, 0.45]. Thus, the equation is different for YDs and for ADs, as it assumes that ADs present a delayed start of DSB repair (e^-0.44*t+0.27^) with respect to YDs (e^-0.44*t^). The basal frequency of DSBs ([n_*foci*]_0_) is also included in the equation because it is different for YDs and ADs (Table 1). Finally, the model estimates that the initial number of γH2AX foci in G1 phase HMECs induced by 1Gy of γ-rays is 𝜃 = 24.32, CI_95%_ = [17.28, 31.35]. Therefore, immediately after irradiation the cells carry those radiation-induced DSBs plus their basal frequency of DSBs ([n_*foci*]_0_) and, according to the model, these γH2AX values are 25.22 for YDs and 26.35 for ADs. Although our model’s estimation of induced DSBs is lower than the 35 DSBs/Gy reported previously [25], it is very similar to others’ estimations of ~25 DSBs/Gy in G1 cells [26,27]. Discrepancies in the number of DSBs induced can be attributed to the source of radiation, the dose rate used in each experiment or to an overestimation of the number of DSBs detected by PGFE methodology.

As shown in Figure 3B, this model renders estimated DSB repair kinetics between 1 and 24h after IR for YDs and ADs that fit well the data observed. Although not strictly applicable, the model has also been used to make an extrapolation corresponding to the kinetics of DSB repair from the time point immediately after irradiation to 1h pIR, shown as dotted colored lines (inset in Figure 3B). The dotted line in YDs reaches the value of 25.22 γH2AX foci at a time close to 0 (blue arrowhead in Figure 3B inset), suggesting that YDs initiate repair immediately after irradiation and they efficiently diminish the number of DSBs during this first hour. Instead, ADs maintain the number of γH2AX foci they had immediately after IR for a longer time, because when data obtained is extrapolated from 1h pIR backward (dotted red line in Figure 3B inset) the value of 26.35 γH2AX foci is attained at a time between 0 and 1h (red arrowhead in Figure 3B inset), suggesting that ADs begin to resolve DSBs later than YDs. Thus, ADs reach the first hour after irradiation carrying more γH2AX foci, which are markers of unresolved DSBs. Hence, it is tempting to speculate that a period of latency exists before ADs are able to fire a fully operative DNA repair response, although once launched, they are able to repair with a speed similar to that of YDs.

## DISCUSSION

To investigate the age-associated impairment of genomic integrity, we examined the DNA-DSB repair efficiency in cells from healthy individuals of different ages. The increased basal frequency of γH2AX foci with donor’s age observed in HMECs is in agreement with results reported in other cell types from healthy human donors [11–14] and reveals an age-dependent accumulation of DSBs. The observed age-related increase in the number of endogenous DSBs could be attributed to a stochastic accumulation of damage with time or alternatively, an alteration of the DSB repair mechanism could account for an accelerated accumulation of unresolved DSBs with age [15–17]. In this regard, our results show that HMECs from ADs presented increased frequencies of DSBs at all times analyzed after IR exposure, which manifests an impaired ability to repair DSBs with age. In fact, the hierarchical clustering analysis performed using data from γH2AX foci scored at all time points, efficiently grouped donors by age, thus demonstrating that analysis of γH2AX foci disappearance after IR exposure could be a potential marker for physiological aging. It is worth to point out that this analysis efficiently unmasked inter-individual variation amongst donors with similar ages, which grouped in an intermediate cluster, and this is especially clear in aged donors. High inter-individual heterogeneity in γH2AX analysis has also been reported in studies measuring γH2AX fluorescent intensity in blood samples [20,28]. Along with inter-individual variability, γH2AX foci disappearance data in HMEC revealed significant inter-cellular variation in the frequency of γH2AX foci in aged donors, which in fact arises as a remarkable feature of ADs. This is in accordance with the recently published results from Cheung and colleagues [29] as they found an age-associated increased cell-to-cell variability and an increased inter-individual heterogeneity in chromatin modifications using a mass cytometry analysis. Efficient tools that allow the detection of donors in which physiological aging does not completely match with chronological aging might be useful to improve and adapt preventive diagnostic controls among other medical procedures.

The repair kinetics we observed in HMECs from older individuals after irradiation is in agreement with the general notion of an age-associated decline in the DNA repair capacity, which is evidenced as a decreased γH2AX foci disappearance after IR exposure in different cell types [14,18,19]. However, an increased DSB repair rate with age has also been reported in blood mononuclear cells from 94 healthy donors [20]. We propose that the apparent disparity among these studies could be explained with our here presented experimental and modeled data. Although ADs show a delay in the initiation of repair, once DSB repair has been initiated, both groups of donors display similar DSB repair kinetics. This delayed firing translates into the accumulation of yet to be resolved DSBs in early times after irradiation. Eventually, ADs launch the repair machinery and they start to resolve these DSBs, appearing as even more efficient than YDs, but only because they have repaired less DSBs immediately before.

A delay in DSB repair initiation could be explained by initial difficulties in loading repair proteins to DSB sites. Primary fibroblasts showed a delayed recruitment of MRE11 and RAD50 proteins with increasing donors’ age [11]. Also, a delayed recruitment to DSB sites of 53BP1 –a repair protein that is involved in the non-homologous end joining (NHEJ) repair pathway– was described in *in vitro* aged HMECs with a time-course experiment of 53BP1 foci formation [10]. In agreement with this, an age-associated decline of the NHEJ repair efficiency was reported in mice [30], rats [31] and human senescent cells [32]. Cell lines defective in ATM or 53BP1 were described as presenting an accumulation of long-lasting residual DSBs [33], suggesting that a defective recruitment could also translate into some kind of repair defect. In line with this, we observed that the repair defect of aged donors is accompanied by an increased frequency of γH2AX foci at 24h after irradiation. Although we cannot rule out that these residual DSBs correspond to complex damage sites or to heterochromatin-located DSBs that are being repaired slowly, we hypothesize that their presence is related to the delay in the firing of the DDR. Long-lasting γH2AX signaling after IR exposure was proposed to be a marker of DNA damage and aging [34] and was correlated with radiosensitivity in mammalian cell lines [35].

A delay in DSB repair initiation, probably related to difficulties in launching an effective DDR, poses a relevant threat to genomic integrity, as the accumulation of unresolved DSBs leads to increasing probabilities of illegitimate repair [36]. Accumulation of genomic rearrangements arising from illegitimate DSB repair was reported in different tissues from old individuals [37]. These abnormalities can affect multiple genes and they are a potential source of oncogenic transformation. It can be of particular concern in individuals exposed to low and protracted doses of radiation in which the repair machinery is continuously challenged. Epidemiological studies have demonstrated an increased excess risk for some types of cancer after exposure to low and protracted doses of radiation with age at exposure [38]. For example, individuals exposed to the radioactive contamination of the Techa River in the Urals had an increased excess relative risk of cancer mortality with increasing age at first exposure [39]. Also, data from workers at the Oak Ridge National Laboratory exposed to low radiation doses revealed an association between age at exposure and cancer mortality [40]. Thus, the impaired ability in DSB repair makes older people among the adult population particularly susceptible to ionizing radiation detrimental effects.

Our study provides valuable information about the relation between aging and DNA-DSB accumulation in human mammary epithelial cells from healthy donors, and we expect our results will serve as a basis for further studies regarding impaired DSB repair mechanisms in aged individuals. Future studies would be necessary to explore the mechanisms responsible for the delay in the initiation of DSB repair with age and their implications in the global cellular context.

## MATERIALS AND METHODS

### Cell culture

Finite lifespan pre-stasis HMECs were obtained from reduction mammoplasty tissue of 11 donors: 48R (16 yo), 240L (19 yo), 168R (19 yo), 184 (21 yo), 59L (23 yo), 123 (27 yo), 153L (60 yo), 112R (61 yo), 122L (66 yo), 29 (68 yo), 429ER (72 yo), or peripheral non-tumor containing mastectomy tissue of 1 donor: 353P (72 yo). Donors were classified into two groups depending on age: young donors (YDs, ≤ 27 years old) and aged donors (ADs, ≥ 60 years old). When referring to donors, the group of age is followed by the specimen identification and the age of the donor in parentheses.

Cells were cultured as pre-stasis strains using M87A medium with cholera toxin and oxytocin according to previously reported methods [21], with the addition of 100 U/ml penicillin and 100 μg/ml streptomycin. Cells were incubated at 37ºC and 5% CO_2_ atmosphere. The passage number indicates each time the cells have been detached form the petri dish using trypsin and seeded into new vessels. The population doubling (PD) indicates each time a cell has divided and was calculated as described by Greenwood and colleagues [41], using the equation: PD = [log(cells harvested/cells plated)]/log2. Cells have been obtained from mammary gland surgical discarded tissues that are subcultured twice before calculation of the PD.

### SA-β-Gal activity detection

SA-β-Gal activity was detected as described by Debacq-Chainiaux [42]. Blue staining was detected under an IX71 microscope equipped with DP20 camera and cell^∧^A software (Olympus, Hamburg, Germany).

### Irradiation

When indicated, exponentially growing HMECs were exposed to 1 Gy of γ-rays using an IBL-437C R-137 Cs irradiator (dose rate of 5.10 Gy/min).

### Immunodetection

### *Flow cytometric analysis*

After trypsinization, HMECs were blocked for 20 minutes in PBS-1% BSA, incubated for 30 minutes with anti-CD227-FITC (clone HMPV, Becton Dickinson, Franklin Lakes, NJ, USA) and anti-CD10-PE (clone HI10a, BioLegend, San Diego, CA, USA) at a final concentration of 1:100, all on ice. Flow cytometric analysis was performed using a FACSCanto (Becton Dickinson).

### *Immunofluorescence*

To detect γH2AX and pericentrin, HMECs were fixed in 4% paraformaldehyde for 15 minutes and permeabilized in a 1xPBS-0.5% Triton- X100 for 20 minutes. To detect γH2AX and claudin-4, cells were fixed with ice-cold methanol for 10 minutes. Cells were incubated for 1 hour with blocking solution (1xPBS-0.1% Tween20-3% FBS) before applying primary antibodies mouse anti-γH2AX (Ser139) (clone JBW301, Millipore, Madrid, Spain), rabbit anti-pericentrin (Abcam, Cambridge, UK) or rabbit anti-claudin-4 (Abcam) at 1:1000, 1:2000 and 1:250 final concentrations respectively. Secondary antibodies anti-mouse Cy3 (Jackson ImmunoResearch Inc., Cambridge, UK) and anti-rabbit A488 (Thermo Fisher Scientific, Waltham, MA, USA) were applied at a final concentration of 1:800 and 1:500 (claudin-4) or 1:1000 (pericentrin) respectively. Nuclei were counterstained with 4’,6-diamidino-2-phenylindole (DAPI) at a final concentration of 0.25 μg/ml. For image acquisition an Olympus BX61 epifluorescent microscope equipped with a CV-M4+CL camera (JAI, Grosswallstadt, Germany) and Cytovision software (Applied Imaging, Newcastle, UK) were used.

### Automated microscopy and γH2AX foci counting

γH2AX foci counting was done following a semi-automatic approach. Images from slides with γH2AX and pericentrin immunofluorescence were captured using an Olympus BX61 epifluorescence microscope equipped with an automatic motorized stage (BX-UCB, Olympus) and a CCD camera (CV-M4+CL, JAI). The capture methodology was adapted from the Spot-counting system (Spot AX software, Applied Imaging) as described by Hernández [24]. Images were acquired automatically with a 60x objective using predefined settings. Four z-stacks were acquired for γH2AX and 6 for pericentrin, with a step size of 1.55 μm between planes. Cells with only one pericentrin signal were selected and γH2AX foci were scored using FociPicker3D algorithm for Fiji software [43].

### Statistical analysis and data modelling

Descriptive analysis and graphics were performed using Microsoft Excel (Microsoft® Excel® 2011, v14.1, Redmond, Washington, USA) and GraphPad Prism 6 (GraphPad Software Inc., San Diego, CA, USA) with methods indicated in the results where applicable. When comparing the number of γH2AX foci/cell among individual donors, Kruskal-Wallis test with Dunn’s multiple comparisons correction was applied and different letters indicate statistical differences (*p*-value < 0.05) between donors in the graphical representation.

In order to statistically compare the two age groups at each time point analyzed, a generalized linear model with a Negative Binomial distribution response and with repeated measures for each donor was established. The estimated values for γH2AX foci number and the corresponding confidence intervals were obtained using SAS software (SAS v9.4, SAS Institute Inc., Cary, NC, USA).

For the hierarchical cluster analysis, standardized values of γH2AX foci from the 12 donors along the four time points (non-irradiated, 1h, 2h and 24h pIR) were used. Standardized data was obtained by subtracting the mean number of each condition (donor, time and replicate) to the number of γH2AX foci scored for each cell and then dividing this value by the standard deviation of the condition. As it was defined by Everitt and colleagues [44], a hierarchical classification consists of a series of partitions, which may run from a single cluster containing all individuals, to n clusters each containing a single individual. In our case we wanted to determine the inter-group (young *vs* old) proximity, and thus the Ward method [45] was applied using R software (version 3.4.4, Vienna, Austria). In this method, the criterion for choosing the pair of clusters to merge at each step is based on the size of the error sum-of-squares. Hierarchical clustering is represented by a two-dimensional diagram known as a dendrogram, which illustrates the fusions or divisions made at each stage of the analysis.

A first order kinetic reaction was established to obtain estimations regarding the kinetics of DSB repair in YDs and ADs. This approach was done using methodologies for Nonlinear Regression Model using SAS software (SAS v9.4, SAS Institute Inc.). The established model is described by the following equation:

n_focit=θ*eβ1*t+n_foci0 if YDθ*eβ1*t+β0+n_foci0 if AD

where [*n_foci*]*_t_* is the number of γH2AX foci at a concrete time after irradiation, 𝜃 is the number of radiation-induced DSBs, *β_1_* is the γH2AX foci decay proportion, [*n_foci*]_0_ is the basal frequency of γH2AX foci (before irradiation) and *β_0_* is a constant of delayed repair onset. This model assumes that (1) the same number of DSBs per unit of radiation are induced in YD and AD cells immediately after irradiation 𝜃; (2) cells do not reach complete repair, but instead they reach the basal frequency [n_*foci*]_0_ of DSBs and (3) cells from ADs suffer a delay in DSBs repair initiation (β_0_).

## Supplementary Material

Supplementary Figures
