# Supplementary material for: Delayed γH2AX foci disappearance in mammary epithelial cells from aged women reveals an age-associated DNA repair defect
Source: Aging (Albany NY). 2019 Mar 14;11(5):1510–23. doi: 10.18632/aging.101849 (PMC6428106; doi:10.18632/aging.101849)
Supplement: Supplementary Figures [file aging-11-101849-s001.pdf]

SUPPLEMENTARY FIGURES

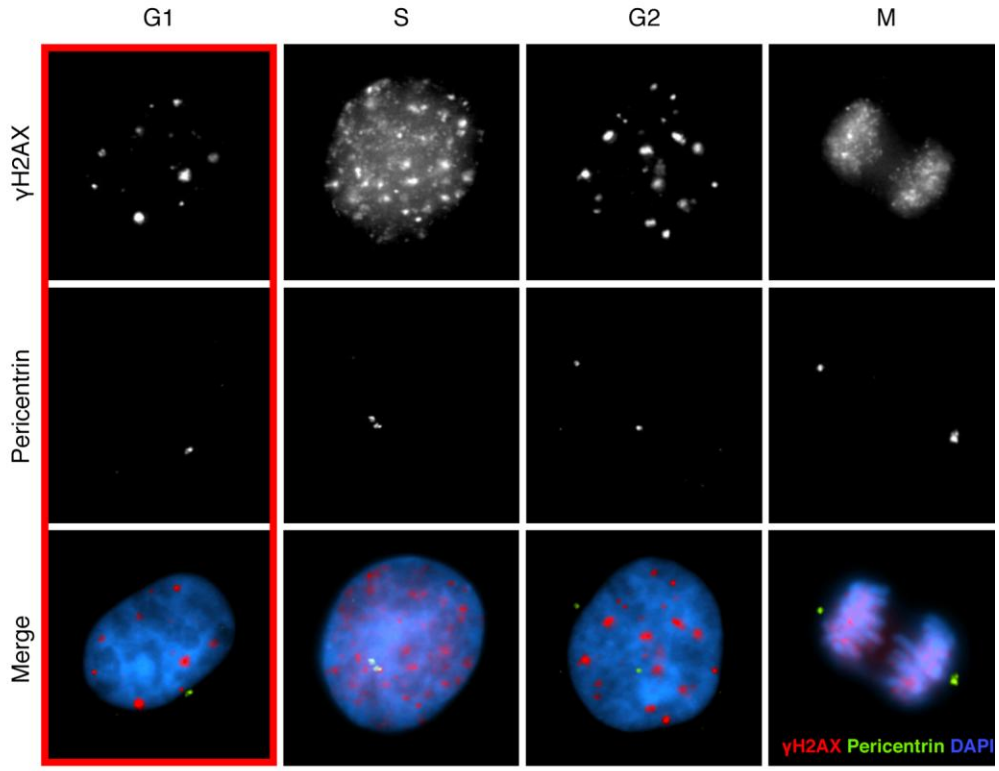

Supplementary Figure 1. Immunofluorescent labeling of γH2AX (Cy3, red), pericentrin (FITC, green) with DAPI (blue) counterstain at different phases of the cell cycle. Scoring of individualized γH2AX foci was restricted to G1 phase cells (red box) by selecting those cells with one pericentrin signal. From late S to mitosis cells have two pericentrin signals. Also the pan-nuclear pattern of γH2AX foci during S phase is very characteristic.

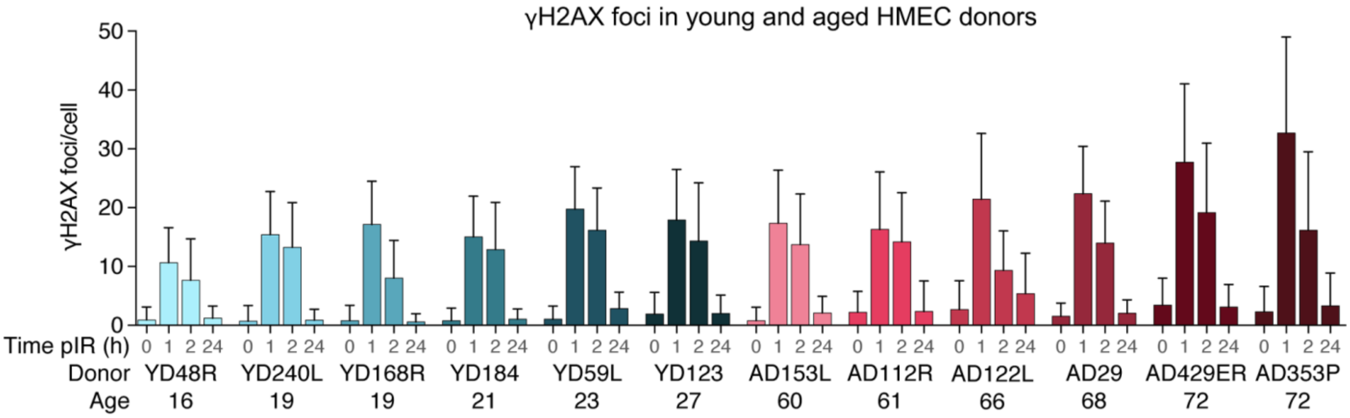

Supplementary Figure 2. Mean number of γH2AX foci per cell and their standard deviation in young and aged HMEC donors before irradiation and at 1, 2 and 24 hours after IR.
